# The Importance of Out-of-Office Blood Pressure Measurement, as Highlighted by the Correlation with Left Ventricular Hypertrophy in an Untreated Hypertensive Population

**DOI:** 10.3390/medicina59091636

**Published:** 2023-09-09

**Authors:** Christina Antza, Georgios Tziomalos, Georgios Kostopoulos, Christina Trakatelli, Vasilios Kotsis

**Affiliations:** 1 3rd Department of Internal Medicine, Aristotle University, Hypertension-24h Ambulatory Blood Pressure Monitoring Center, Papageorgiou Hospital, 56429 Thessaloniki, Greece; 2 1st Cardiology Department, AHEPA University Hospital, Aristotle University of Thessaloniki, 54124 Thessaloniki, Greece

**Keywords:** blood pressure, hypertension, phenotypes, dipping status, left ventricular hypertrophy

## Abstract

*Background and Objectives*: Hypertensive heart disease, especially left ventricular hypertrophy (LVH), is considered to be one of the main types hypertension-mediated organ damage. Hence, the purpose of this study was to examine which method of measuring BP (office BP measurement (OBPM), 24 h ambulatory BP monitoring (ABPM), or home BP monitoring (HBPM)), can be better correlated with echocardiographic LVH in the untreated hypertensive population. *Materials and Methods*: This study’s population consisted of 202 patients 58 ± 15 years old (40.8% males). All patients reported elevated home BP measurements for at least 3 months, but they had never been treated before for hypertension. Office and out-of-office BP measurements, including ABPM on a usual working day and seven-day HBPM, as well as 2D echocardiography, were performed. *Results*: In the univariate analysis, LVH was associated (*p* < 0.05) with a mean 24 h systolic BP (OR: 1.93, CI: 1.29–2.91), a mean 24 h diastolic BP (OR: 1.30, CI: 1.16–1.80), ambulatory daytime systolic (OR: 1.11, CI:1.01–1.82) and diastolic BP (OR: 1.13, CI:1.09–1.17), ambulatory nighttime systolic BP (OR: 2.11, CI: 1.04–4.31), and mean home systolic BP (OR: 1.05, CI:1.01–1.12). Pearson’s correlation analysis showed a significant correlation between the LV mass index and the mean 24 h systolic BP (r = 0.58, *p* < 0.05), daytime systolic BP (r = 0.59, *p* < 0.05), and nighttime systolic BP (r = 0.57, *p* < 0.05). Most of the population with confirmed LVH presented confirmed hypertension (based on ABPM, 48.1% or HBPM, 40%). The second most dominant phenotype was masked hypertension (ABPM, 32.7% and HBPM, 23.7%). The majority (59.3%) had non-dipping status, 20.4% had a reverse dipping pattern, 13% had a dipping pattern, and only 7.3% had extreme dipping BP. *Conclusions*: Out-of-office BP measurement devices seemed to be superior compared to in-office. This advantage is highlighted by better correlations in the identification of LVH as well as the diagnosis of masked hypertension, a condition also highly correlated with LVH.

## 1. Introduction

Hypertensive heart disease, especially left ventricular hypertrophy (LVH), but also systolic and diastolic dysfunction as well as their clinical outcomes, is considered to be one of the main types of hypertension-mediated organ damage [1]. LVH in hypertension is caused as a response to the external stimulation of pressure and volume overload. The increased heart workload results in remodeling and an increase in the left ventricular myocardial mass, in parallel with wall thickening [2]. Although cardiac magnetic resonance is the gold standard for the assessment of this cardiac anatomical and functional assessment [3], due to its high cost and need for specified medical staff, a two-dimensional (2D) echocardiography has been established for patients with hypertension [1].

The prevalence of LVH is high in hypertensives, with an almost 7% prevalence in pre-hypertension and 18% in hypertension, as highlighted by the PAMELA study [4]. A recent meta-analysis showed that LVH is also present in 30% of children and adolescents with primary hypertension [5]. Furthermore, LVH contributes to the manifestation of cardiovascular mortality and morbidity. Hypertensive left ventricular remodeling has been correlated with a 2.5 times higher incidence of cardiovascular events compared to normal geometry, and an almost 5% higher cardiovascular mortality [6,7]. Taking all of these into account as well as the high prevalence of hypertension, the early identification of high-risk patients is of major importance.

Even though LVH is a well-studied target organ damage of hypertension, it is still not clearly answered which method of measuring blood pressure (BP) can better predict LVH. To our knowledge, few data are so far available. Specifically, the data are limited mainly to studies comparing only one of the out-of-office BP measurements with clinic BP [8,9]. Other studies have evaluated both treated and untreated populations [10,11], without taking into account that the effect of antihypertensive agents may change the results. Also, there is evidence based on electrocardiographic [9] but not echocardiographic changes for the evaluation of left ventricular hypertrophy. Finally, some studies have not measured home BP based on the current guidelines [12,13,14].

Hence, the purpose of this study was to examine which method of measuring BP (office BP measurement (OBPM), 24 h ambulatory BP monitoring (24 h ABPM), or home BP monitoring (HBPM)) can be better correlated with echocardiographic LVH in the untreated hypertensive population. The secondary aim was to analyze the prevalence of different BP phenotypes in untreated hypertensive patients with LVH.

## 2. Materials and Methods

### 2.1. Study Population

The population of this cross-sectional study consisted of 202 individuals who attended the Hypertension 24 h ABPM Center of Excellence in the 3rd Department of Internal Medicine at the Aristotle University of Thessaloniki in Greece, from 2018 to 2020. All patients included in this analysis reported elevated BP measurements at home for at least 3 months, but they had neither been treated before for hypertension nor during the study. The participants were extensively informed about the procedure and gave their informed consent to take part in this study. Patients aged less than 16 years old were excluded from the study, as were patients who did not complete all types of BP measurements. Other exclusion criteria were previous cardiovascular disease, secondary hypertension, end-stage renal disease, and concomitant systematic or inflammatory diseases. The study was approved by the Ethics Committee of the Medical School at the Aristotle University of Thessaloniki in Greece.

### 2.2. Blood Pressure Measurements

Three different types of BP monitoring (OBPM, HBPM, 24 h ABPM) were performed on all participants during the study. All measurements were performed following the latest European (ESH/ISH) guidelines [1].

Regarding OBPM, it was measured three times in the arm with higher values of BP for two consecutive days by the same investigator (CA), using a mercury sphygmomanometer. The average of these six measurements was taken into account as the office BP. The participants underwent also 24 h ABPM (Spacelabs 90217, Spacelabs Inc., Redmond, WA, USA). The appropriate size cuff was placed around the nondominant arm, and three measurements were made, along with sphygmomanometric measurements to verify that the average of these values did not differ more than 5 mmHg. The 24 h ABPM is programmed to measure the BP every 15 min/hour during the day, and 20 min/hour during the night, in order to ensure at least 80 successful recordings. The times specifying the daytime and nighttime were fixed (8.00 a.m. to midnight for daytime, and midnight to 7.59 a.m. for nighttime), but in case of extreme differences (for example, shift workers), the times were changed manually based on the patient’s self-reports. Finally, HBPM was performed with the use of the WatchBP Home device (Microlife AG Swiss, Widnau, Switzerland) for 7 consecutive days. The patients were informed on how to use the HBPM by the principal investigator (CA). The device was set up to measure BP twice daily during a specific period of time. Two measurements were performed in the morning (06:00–09:00) and two in the evening (18:00–21:00), with an interval of 1 min. The HBPM was taken into account as the average of the BP records, excluding the BP measurements from the first day. The daytime BP was taken into account as the average of the morning BP recordings, and the nighttime BP as the average of the evening BP recordings, all after excluding the measurements from the first day.

### 2.3. Blood Pressure Phenotypes

According to the ESC/ESH latest guidelines [1,15], the study population was categorized into four phenotypes by combining the cut-off values for the OBPMs and daytime ABPMs. Specifically, those classified as true normotensive subjects presented OBPMs of less than 140/90 mmHg and ABPMs of less than 135/85 mmHg, and true hypertensive subjects presented OBPMs equal to or higher than 140/90 mmHg and ABPMs equal to or higher than 135/85 mmHg. Patients were categorized as white-coat hypertensives (WCHT) if they presented OBPMs equal to or higher than 140/90 mmHg and ABPMs  less than 135/85 mmHg, and as masked hypertensives (MHT) when their OBPMs were less than 140/90 mmHg and their ABPMs were equal to or higher than 135/85 mmHg. Similarly, the participants were categorized based on the cut-off values for OBPMs and HBPMs, with HΒPM values less than 135/85 mmHg considered normal.

Regarding the BP pattern during the nighttime [1,16], the patients were divided into two categories: dippers, when the night BP average fell by more than 10% compared to the daytime BP average, and non-dippers, when there was less than a 10% reduction, based on the ABPM. As further subcategories of the dipping and non-dipping status, we also divided patients into two further groups: extreme dippers were those presenting > 20% nighttime BP reduction, and reverse-dippers were those presenting a nighttime BP higher than the daytime BP average.

### 2.4. Echocardiogram

All subjects underwent complete 2D transthoracic echocardiography according to the current guidelines [17]. Images were obtained in the parasternal long- and short-axis views and apical two- and four-chamber views. Echocardiography was performed by an experienced cardiologist/sonographer (GT), using high-end ultrasound system (Vivid E95, GE Healthcare, Chicago, IL, USA). The measurements were performed on more than one cardiac cycle, accounting for interbeat variability. The echocardiographic data were stored in a cine-loop format for offline analysis.

The left ventricular mass index was calculated with the linear method recommended by the American Society of Echocardiography/European Association of Cardiovascular Imaging (ASE/EACVI) as follows: left ventricular mass index = 0.8 × 1.04 [(IVS + LVID + PWT)^3^− (LVID)^3^] + 0.6, where IVS is the interventricular septum, LVID is the left ventricular internal diameter, and PWT is the posterior wall thickness [18]. Linear measurements of the left ventricular diameter, interventricular septum, and left ventricular posterior wall thickness were derived directly from the 2D echocardiographic parasternal views, perpendicular to the left ventricular long axis, and measured at the level of the mitral valve leaflet tips. Images were acquired at the end-diastole, and measurements were made at the tissue–blood interfaces [18]. The indexation of the left ventricular mass to the height raised to the allometric power of 2.7 (LVM/height^2.7^) was used over indexing to BSA, since it demonstrates better predictive value for cardiovascular outcomes and the better detection of obesity-related LVH [19,20]. Finally, in accordance with recommendations, the upper reference limits of a normal left ventricular mass index by linear measurements are 47 g/m^2.7^ for female patients and 50 g/m^2.7^ for male patients [15].

### 2.5. Statistical Analysis

SPSS 25.0 (SPSS Inc., Chicago, IL, USA) was used to statistically analyze the data. Continuous variables are reported as the mean ± SD, and categorical variables as counts and percentages. The normality was evaluated with the Kolmogorov–Smirnov or the Shapiro–Wilk test, as appropriate. Left ventricular hypertrophy was analyzed also as a categorical variable, dividing patients as those presenting or not LVH. Crosstabs and Chi-tests were used to measure the differences between the different hypertension phenotypes and the existence of LVH. Logistic regression analysis was also performed to evaluate how different BP parameters affected LVH. Finally, Pearson’s correlation coefficient was used to examine the relationships and correlations between different continuous BP variables and the continuous variable of the left ventricular mass index.

## 3. Results

### 3.1. Descriptive Statistics

Our population consisted of 202 patients aged 58 ± 15 years old, of which 40.8% were males. Of them, 44% had a history of dyslipidemia, and 16.9% had diabetes mellitus type 2. Only one-fifth of the population reported smoking (21.8%) and drinking alcohol (19.7%), while there was a trend in the population of being overweight. A total of 36.8% presented a normal left ventricular size according to the 2D echocardiography assessment, while the rest had signs of LVH. The mean systolic BP was higher in the OBPMs (145.6 ± 17.9 mmHg) compared to the HBPMs (140 ± 14.8 mmHg) and 24 h ABPMs (129.9 ± 15.7 mmHg). Similarly, higher values of diastolic BP were observed in the OBPMs (85.2 ± 12.3 mmHg) compared to the HBPMs (83.1 ± 11.3 mmHg) and 24 h ABPMs (77.2 ± 11.7 mmHg). Descriptive statistics of our population are provided in Table 1.

### 3.2. Univariate Logistic Regression Analysis of Left Ventricular Hypertrophy using Different Blood Pressure Measurements

In the univariate analysis, LVH showed significant associations (*p* < 0.05) with almost all parameters from the ABPMs, and specifically with the mean 24 h systolic BP, mean 24 h diastolic BP, daytime systolic and diastolic BP, and nighttime systolic BP. Regarding the HBPM, LVH showed significant associations (*p* < 0.05) only with the mean systolic BP. No relationship was found for the mean diastolic home BP, daytime and nighttime home systolic, or diastolic BP. Finally, office BP measurements showed no association (*p* ≥ 0.05). The results regarding the univariate analysis are provided in Table 2.

### 3.3. Pearson Correlation between Left Ventricular Mass Index and Different Blood Pressure Parameters

Pearson’s correlation analysis (Table 3) showed a significant positive correlation between the left ventricular mass index and the mean 24 h systolic BP (r = 0.58, *p* < 0.05), daytime systolic BP (r = 0.59, *p* < 0.05), and nighttime systolic BP (r = 0.57, *p* < 0.05). Regarding the HBPM, there were weak positive correlations between the left ventricular mass index and the mean systolic BP (r = 0.29, *p* < 0.05), daytime systolic BP (r = 0.28, *p* > 0.05), and nighttime systolic BP (r = 0.22, *p* < 0.05). No other significant correlations were revealed with the other BP parameters.

### 3.4. Prevalence of Different Blood Pressure Phenotypes in Patients with Left Ventricular Hypertrophy

Most of our population with confirmed LVH presented confirmed hypertension either evaluated by the combination of the OBPM and 24 h ABPM (48.1%) or by the combination of the OBPM and HBPM (40%). The second most dominant phenotype in this population was masked hypertension (32.7%, based on 24 h ABPM, and 23.7%, based on HBPM). Taking into account the 24 h ABPM, 11.5% presented white-coat hypertension, and 7.9% normotension. Taking into account the HBPM, the prevalence of white-coat hypertension and normotension was almost equal (18.4% and 17.9%, respectively).

Regarding the dipping status of the population during sleep, the majority (59.3%) had a non-dipping status, 20.4% had a reverse dipping pattern, 13% had a dipping pattern, and only 7.3% had an extreme dipping BP. The dipping status was evaluated only by the 24 h ABPM. The results regarding the BP phenotypes are presented in Table 4.

## 4. Discussion

This study shows the importance of out-of-office BP evaluation in the identification of LVH in the untreated hypertensive population. Systolic and diastolic BP from ABPM and systolic BP from HBPM were found to be correlated with LVH. Out-of-office BP showed superiority, as the OBPM parameters revealed no correlation. In patients with hypertension and LVH, the most dominant phenotype was confirmed hypertension, and the second most dominant was masked hypertension, defined either by ABPM or HBPM. Masked hypertension and normotension presented lower prevalence. Regarding the dipping status, more than half of the population presented a non-dipping status.

To the best of our knowledge, data regarding the untreated hypertensive population are limited, and the currently existing data do not provide a clear answer of which method is best correlated with LVH. Stergiou G et al. [21] showed, for the first time, a correlation between systolic HBPM and ABPM and the left ventricular mass index of 68 untreated hypertensive patients. However, the correlation was weak for both the ABPM (correlation coefficient: 0.23, *p* < 0.05) and HBPM (correlation coefficient: 0.31, *p* < 0.01). This weak correlation could be due to the small number of participants in the trial. Similarly, Her A-Y et al. [22] evaluated 93 untreated hypertensive patients and also showed that systolic ABPM and HBPM could be correlated with the left ventricular mass index, contrary to the OBPM parameters. Similar to our results, systolic ABPM presented a stronger correlation (correlation coefficient: 0.42, *p* < 0.05) compared to systolic HBPM (correlation coefficient: 0.33, *p* < 0.05). Evaluating an untreated hypertensive population consisting of 408 adults, Schwartz et al. showed that the mean home SBP (0.486) provided a statistically significant better correlation with LVH not only compared to OBPM (0.365, *p* < 0.001) but also compared to ABPM (0.394, *p* < 0.001). In regard to DBP, there were not any statistically significant differences among HBPM, OBPM, and ABPM [23]. Finally, a recently published study with a mixed population (either receiving or not antihypertensive treatment), consisting of more than 900 patients, showed that for each 10 mmHg increase in systolic HBPM, the left ventricular mass increased by 10 g/m^2^ (95% CI; 3.7–27, *p* = 0.01), and for 24 h systolic ABPM, it increased by 2.3 g/m^2^ (95% CI 0.76–3.9, *p* < 0.01) [10].

Regarding the phenotype of hypertension in naïve patients and its correlation with LVH, the data are limited to patients already divided into having only MHT or WCHT and into treated and untreated patients. In a recent publication by Inanc I.H et al., patients with MHT presented almost 25% more LVH in the echocardiography examination. This percentage was found to be much lower (2%) in untreated WCHT, as revealed by a big data population study [24]. To the best of our knowledge, there is only one study similar to our analysis. Specifically, in 120 patients with LVH without other known causes, MHT was detected in a statistically significant higher percentage (28.3%) compared to the controls (6.6%) [25]. The percentage of MHT is different from our results (36.7%, based on 24 h ABPM), but quite similar to normotension (8.3%, based on 24 h ABPM). However, as there is no reference for the other hypertension phenotypes (WCHT and hypertension), these associations may not be reliable. Data regarding the prevalence of phenotypes defined by HBPM in untreated LVH patients are not available in the literature.

The present study was designed as cross-sectional and therefore, causation cannot be determined for any of the observed relationships. Hence, the question of whether the hypertensive phenotypes and the dipping status cause LVH or the opposite cannot be answered. Furthermore, the population consisted only of Caucasians, and therefore, the results cannot be generalized to other races. Finally, due to the small sample (presenting LVH), ROC curve analysis could not be performed in order to investigate which BP measurement method can better diagnose LVH. On the other hand, the strengths of the current study are the analysis of an untreated population, the use of 2D echocardiography instead of electrocardiography, and the fact that the BP measurements were performed based on the ESH guidelines [26].

In clinical practice, based on our results, it seems of major importance to use one of the out-of-office BP measurements in untreated patients, at least when they are referred for the first time to a hypertension clinic, as the OBPM cannot be correlated with LVH. Furthermore, the use of out-of-office devices at the first visit seems also to be important in order to exclude masked hypertension, as it seems to be correlated with already established LVH. Taking into account the cost effect, out-of-office BP measurements could be limited to high-risk populations for presenting masked hypertension. This population is typically a young-age population that reports alcohol consumption or smoking and increased activity or increased work stress [26]. An OBPM of 130/80 mmHg seems also to provide the best sensitivity and specificity for the diagnosis of masked hypertension [27]. Based on the reported results, ABPM seems to have superiority compared to HBPM, as it presented better correlations with LVH but also it could identify the variance of hypertension overnight. As the majority of hypertensive patients with already established LVH do not present an ideal dipping status, ABPM could help clinicians identify which status their patients have and select the best option of antihypertensive therapy (long-acting effect or, if needed, intermediate-acting used in the evening), according to this status.

Even the latest ESH guidelines [15] still use only OBPM to define hypertension. The results of this study may indicate the need for at least one of the out-of-office BP measurements for the definition of hypertension, as its importance is high due to its correlation with target organ damage. Furthermore, the results of this study are in accordance with and further support the latest ESH guidelines [15] in regard to the indications for ABPM or HBPM. Specifically, some of the main indications are normal OBPMs in patients presenting target organ damage as well as the identification of WCHT or MHT. Future research goals are to investigate if any of the BP measurement methods—from the first visit—can predict LVH in the future or if those with already established LVH have better outcomes when follow-up is based on out-of-office BP measurements.

## 5. Conclusions

To conclude, out-of-office BP measurement devices seem to be superior compared to OBPM. This advantage is highlighted by better correlations with the identification of LVH as well as masked hypertension conditions, which are also highly correlated with LVH.

## Figures and Tables

**Table 1:** Demographics of the study population.

Variables	Mean ± SD
Age (years)	58.0 ± 15
Sex (male %)	40.8
BMI (kg/m^2^)	29.8 ± 5.7
eGFR (ml/min/1.73 m^2^)	85.6 ± 21.7
Dyslipidemia (%)	44
Diabetes mellitus (%)	16.9
Current smokers (%)	21.8
Alcohol (yes%)	19.7
Mean 24 h SBP (mmHg)	129.9 ± 15.7
Mean 24 h DBP (mmHg)	77.2 ± 11.7
Mean home SBP (mmHg)	140 ± 14.8
Mean home DBP (mmHg)	83.1 ± 11.3
Mean office SBP (mmHg)	145.6 ± 17.9
Mean office DBP (mmHg)	85.2 ± 12.3
LV hypertrophy (%)	36.8

BMI: body mass index, eGFR: estimated glomerular filtration rate, SBP: systolic blood pressure, DBP: diastolic blood pressure, LV: left ventricular.

**Table 2:** Univariate logistic regression analysis of different BP parameters.

Variable	OR	95% Cl	*p*
Based on 24 h ABPM			
Mean 24 h SBP	1.93	1.29–2.91	<0.05
Mean 24 h DBP	1.30	1.16–1.80	<0.05
Daytime SBP	1.11	1.01–1.82	<0.05
Daytime DBP	1.13	1.09–1.17	<0.05
Nighttime SBP	2.11	1.04–4.31	<0.05
Nighttime DBP	0.45	0.19–1.07	>0.05
Based on HBPM			
Mean SBP	1.05	1.01–1.12	<0.05
Mean DBP	1.23	0.38–3.93	>0.05
Daytime SBP	0.93	0.57–1.49	>0.05
Daytime DBP	1.32	0.67–2.59	>0.05
Nighttime SBP	1.07	0.72–1.57	>0.05
Nighttime DBP	0.81	0.45–1.44	>0.05
Based on OBPM			
Mean office SBP	1.03	0.99–1.08	>0.05
Mean office DBP	1.02	0.94–1.09	>0.05

ABPM: ambulatory blood pressure monitoring, SBP: systolic blood pressure, DBP: diastolic blood pressure, HBPM: home blood pressure monitoring, OBPM: office blood pressure measurement.

**Table 3:** Pearson’s correlations between different BP parameters and left ventricular mass index.

Variable	r	*p*
Based on 24 h ABPM		
Mean 24 h SBP	0.58	<0.05
Mean 24 h DBP	−0.07	>0.05
Daytime SBP	0.59	<0.05
Daytime DPB	−0.1	>0.05
Nighttime SBP	0.57	<0.05
Nighttime DBP	0.01	>0.05
Based on HBPM		
Mean SBP	0.29	<0.05
Mean DBP	0.07	>0.05
Daytime SBP	0.28	>0.05
Daytime DBP	0.07	>0.05
Nighttime SBP	0.22	<0.05
Nighttime DBP	0.06	>0.05
Based on OBPM		
Mean office SBP	0.07	>0.05
Mean office DBP	−0.04	>0.05

ABPM: ambulatory blood pressure monitoring, SBP: systolic blood pressure, DBP: diastolic blood pressure, HBPM: home blood pressure monitoring, OBPM: office blood pressure monitoring.

**Table 4:** Prevalence of different blood pressure phenotypes in patients with left ventricular hypertrophy.

	Different BP Phenotypes			
Within Population Presenting LV Hypertrophy	Based on 24 h ABPM			
	Normotension	White-Coat	Masked	Hypertension
	7.9%	11.5%	32.7%	48.1%
	Based on HBPM			
	Normotension	White-Coat	Masked	Hypertension
	17.9%	18.4%	23.7%	40%
	Dipping status during night			
	Extreme Dipping	Dipping	Non-Dipping	Reverse
	7.3%	13%	59.3%	20.4%

LV: left ventricular, BP: blood pressure, ABPM: ambulatory blood pressure monitoring, HBPM: home blood pressure monitoring.

## Data Availability

The data presented in this study are available on request from the corresponding author.
